# Acetaminophen Toxicity

**DOI:** 10.21980/J8435R

**Published:** 2025-01-31

**Authors:** Rachel Whittaker, Navneet Cheema

**Affiliations:** * University of Chicago, Department of Emergency Medicine, Chicago, IL

## Abstract

**Audience:**

This is a practice structured interview case which is appropriate for emergency medicine residents at all levels of training.

**Introduction:**

Acetaminophen (APAP) is an over-the-counter medication commonly used by adult and pediatric populations. While acetaminophen has demonstrated to be reasonably safe and well-tolerated at therapeutic doses, it can cause severe hepatic toxicity if taken in excess. Acetaminophen toxicity is the most common cause of acute hepatic failure in the United States, accounting for approximately 50 percent of all reported cases and 20 percent of liver transplants.^1^ In 2021, poison control received more than 80,000 cases involving acetaminophen-containing products, and acetaminophen toxicity is responsible for 56,000 – 75,000 emergency department visits annually.^2^,^3^ Acetaminophen toxicity is compounded by introduction of acetaminophen combination products, with unintentional and chronic overdose accounting for over 50 percent of cases of acetaminophen-related acute hepatic failure in the United States and United Kingdom.^4^ Given the prevalence of acetaminophen toxicity and oftentimes vague presentation of symptoms, it is imperative that emergency medicine physicians promptly identify and manage acetaminophen toxicity.

**Educational Objectives:**

At the end of this practice oral board session, examinees will be able to: 1) demonstrate an ability to obtain a complete medical history in an oral boards structured interview format, 2) review appropriate laboratory tests and imaging to evaluate abdominal pain, 3) investigate a broad differential diagnosis for right upper quadrant abdominal pain, 4) recognize chronic acetaminophen toxicity, 5) initiate the appropriate treatment for chronic acetaminophen toxicity, 6) demonstrate effective communication with the patient, consultants, and the admitting team.

**Educational Methods:**

This is a structured interview case intended to evaluate learner thought processes throughout the evaluation, workup, and diagnosis of a patient with acetaminophen toxicity.

**Research Methods:**

The practice structured interview case was developed and then tested in a small group environment with emergency medicine residents at different levels of training. After the case was completed, learners and instructors were given the opportunity to assess its strengths and weaknesses by providing electronic feedback during a residency conference. The format of oral boards’ assessing the strengths and weaknesses of a case was mimicked through having one instructor to one to two residents per case administration. Subsequent modifications were made to remove ambiguity based on the feedback provided.

**Results:**

This case was administered as part of our residency oral boards didactics series. Thirty-one EM residents PGY1-PGY3 were administered in the case. Ten learners completed an evaluation form as part of the annual Program Evaluation Committee survey. When asked to evaluate the quality of the oral boards didactics series on a scale of 1 to 5, they rated it a 4.4 out of 5.

**Discussion:**

This practice structured interview case was an effective model to help prepare residents for their oral boards and to assess their understanding of identifying and treating acetaminophen toxicity. Based on learner and instructor feedback, several laboratory results were added to the stimulus package and clarifying language to the examiner script.

**Topics:**

Oral boards, structured interview, abdominal pain, acetaminophen toxicity.

## USER GUIDE

[Table t1-jetem-10-1-si1]

**Table t1-jetem-10-1-si1:** 

List of Resources:	
Abstract	1
User Guide	3
For Examiner Only	5
Oral Boards Assessment	11
Stimulus	12

**Learner Audience:**

Interns, Junior Residents, Senior Residents

**Time Required for Implementation:**

Case: This case is designed to be a structured interview case which is allotted 15 minutes per American Board of Emergency Medicine (ABEM) standard.

Debriefing: About 5 minutes per case.

**Learners per instructor:** Ideal is one learner per instructor, can pair residents if needed.

**Topics:**

Oral boards, structured interview, abdominal pain, acetaminophen toxicity.

**Objectives:**

At the end of this oral board session, examinees will be able to:

1. Demonstrate an ability to obtain a complete medical history
2. List the appropriate laboratory tests and imaging to evaluate abdominal pain
3. Investigate a broad differential diagnosis for abdominal pain
4. Recognize chronic acetaminophen toxicity
5. Initiate the appropriate treatment for acetaminophen toxicity
6. Demonstrate effective communication with the patient, consultants, and the admitting team.
7. intensive care unit after consulting with critical care specialists, understanding the need for the complex management of these patients.

### Linked objectives, methods and results

This structured interview oral boards case was designed to teach about acetaminophen toxicity through active resident learning. The case was structured to have the learner simulate real-life emergency medicine practice and progress through patient care by obtaining timely history (Objective 1). After history acquisition, learners must order and interpret relevant labs and imaging (Objective 2). Given that clinical manifestations of acetaminophen overdose are initially non-specific, learners must form a broad differential diagnosis for abdominal pain (Objective 3). Based on information and data provided by instructors, which point to chronic acetaminophen toxicity, learners should hone in on the diagnosis (Objective 4). After identifying the appropriate diagnosis, learner should order appropriate interventions (Objective 5) and disposition (Objective 6) for chronic acetaminophen toxicity.

### Recommended pre-reading for instructor

The instructor should pre-read the examiner instructions prior to the session to familiarize themselves with the case and the structured interview format of oral boards.

### Results and tips for successful implementation

This case is written to prepare learners for emergency medicine oral boards examination. Faculty instructors administered oral boards during resident’s regularly scheduled conference. A matrix was set to span 150 minutes of conference time, where residents either had the case administered individually or in pairs, depending on instructor to learner ratio. Residents rotated through three unique cases that were administered via faculty instructors in person. Residents had 15 minutes to complete the case and 5 minutes to provide feedback immediately after case completion.

This case was administered as part of our residency oral boards didactics series. This specific oral board case was piloted during EM didactics on January 25, 2024. Thirty-one EM residents PGY1-PGY3 were given the case. Ten learners completed an evaluation form as part of the annual Program Evaluation Committee survey. When asked to evaluate the quality of the oral boards didactics series on a scale of 1 to 5, they rated it a 4.4 out of 5. The Likert scale was defined as follows: 1 (unsatisfactory), 2 (below average), 3 (average), 4 (above average), 5 (outstanding). Both the residents and faculty facilitators were emailed for feedback/suggestions regarding the case. All feedback was integrated into this final version of the case.

## Figures and Tables

**Table t2-jetem-10-1-si1:** 

**I. History**				
**1**	Yes	No	(circle)	Obtain which OTC medication the patient took
**2**	Yes	No	(circle)	Obtain personal history of gallbladder disease
**3**	Yes	No	(circle)	Ask social history
**4**	Yes	No	(circle)	Rationale for PROMPT 2 #1
**5**	Yes	No	(circle)	Rationale for PROMPT 2 #2
**II. Physical Examination**				
**5**	Yes	No	(circle)	Perform an abdominal exam
**6**	Yes	No	(circle)	Perform a cardiac exam
**7**	Yes	No	(circle)	Evaluate for fluid status/dehydration
**8**	Yes	No	(circle)	Rationale for PROMPT 4
**III. Differential Diagnosis**				
**9**	Yes	No	(circle)	Diagnosis 1
**10**	Yes	No	(circle)	Diagnosis 2
**11**	Yes	No	(circle)	Diagnosis 3
**IV. Diagnosis Studies**				
**12**	Yes	No	(circle)	CBC
**13**	Yes	No	(circle)	CMP
**14**	Yes	No	(circle)	APAP level
**15**	Yes	No	(circle)	Rationale for PROMPT 7
**V. Treatment and Other Actions**				
**17**	Yes	No	(circle)	NAC
**18**	Yes	No	(circle)	Fluids
**VI. Final Diagnosis**				
**19**	Yes	No	(circle)	Acetaminophen toxicity
**VII. Disposition**				
**20**	Yes	No	(circle)	Admit
**VIII. Transitions of Care**				
**21**	Yes	No	(circle)	Explain the diagnosis to the patient
**22**	Yes	No	(circle)	Provide sign-out to the admitting team

**Table t3-jetem-10-1-si1:** 

**#1**	**Patient Information Form**
**#2**	**CBC**
**#3**	**CMP**
**#4**	**Coagulation panel**
**#5**	**Urine drug screen, Serum alcohol**
**#6**	**APAP, salicylate level**
**#7**	**Right upper quadrant ultrasound (US)**

**Table t4-jetem-10-1-si1:** 

**Age:**	**27**
**Gender:**	**Female**
**Method of Arrival:**	**Private vehicle**
**Chief Complaint:**	**Right upper quadrant abdominal pain**
**Person Providing History:**	**Patient**

**Table t5-jetem-10-1-si1:** 

**General Appearance/History of Present Illness:**	**Patient has been having right upper quadrant abdominal pain since yesterday. She has also been vomiting today. States she recently had a severe migraine and has had to take a lot of over-the-counter medication.**

**Table t6-jetem-10-1-si1:** 

**Vital Signs:**	
**Temp:**	**96.6° F**
**BP:**	**115/75**
**P:**	**114**
**RR:**	**18**
**Pulse Ox:**	**100% on room air**

**Table t7-jetem-10-1-si1:** 

WBC	5.4 × 10^3^/μL
Hgb	12.0 g/dL
Hct	36%
Platelets	173 × 10^3^/μL

**Table t8-jetem-10-1-si1:** 

Na	142 mEq/L
K	3.4 mEq/L
Cl	92 mEq/L
CO2	22 mEq/L
BUN	16 mg/dL
Cr	0.9 mg/dL
Glucose	86 mg/dL
ALT	194 Units/L
AST	137 Units/L
Alk phos	104 Units/L
Serum bilirubin	3.4 mg/dL (total)
	0.3mg/dL (direct)

**Table t9-jetem-10-1-si1:** 

PTT:	33.8 seconds
PT:	15.1 seconds
INR:	1.2

**Table t10-jetem-10-1-si1:** 

**Urine Drug Screen:**	
Ethanol Screen:	None Detected
Amphetamines:	None Detected
Barbiturates:	None Detected
Benzodiazepines:	None Detected
Cocaine:	None Detected
Opiates:	None Detected
Phencyclidine (PCP):	None Detected
Methadone:	None Detected
Oxycodone Screen:	None Detected
Fentanyl, Urine	None Detected

**Table t11-jetem-10-1-si1:** 

**Serum Alcohol:**	<25 mg/dL

**Table t12-jetem-10-1-si1:** 

Acetaminophen:	327 μg/mL
Salicylates:	<1 mg/dL

## FOR EXAMINER ONLY

### Standardized Interview Case Summary

**Diagnosis: Acetaminophen toxicity**

**Case Summary:** 27-year-old female, with no significant past medical history, presenting to the ED via private vehicle for evaluation of right upper quadrant (RUQ) abdominal pain since yesterday. Patient had recently had a migraine and taken an over-the-counter medicine (acetaminophen) for her pain. She has been taking several handfuls over many days and presents as a chronic acetaminophen ingestion with non-bloody, non-bilious emesis.

**Past Medical History (PMH):** No known medical problems

**Past Surgical History (PSH):** None

**Medications:** No prescription medications. Recent acetaminophen use.

**Allergies:** No known drug allergies.

**Family and social history:** No alcohol use. No illicit substances. No significant family history.

**Synopsis of Physical Exam:**

T 98.6° F Oral, BP 115/75, HR 114, RR 18, Pulse Ox: 100% on RA.

Patient appears uncomfortable secondary to pain with scleral icterus, jaundiced skin, dry mucous membranes, and tenderness to palpation (TTP) in right upper quadrant of abdomen.

### Examiner Script

**Case Introduction:**

“Hello Doctor, this is a structured interview case. There is no role playing. In response to the questions I will ask, please give me a LIST of information you would gather to come to a final diagnosis. At times, I may interrupt you to move you through the case; this is not a reflection of your performance You will have 15 minutes to complete the case. Before we begin, do you have any questions?”

“The patient will be a 27-year-old female with vomiting and right upper quadrant abdominal pain.”

Provide Learner Stimulus #1

**HISTORY**

Prompt 1:

“Here is the initial information regarding this patient. After you have read it, please give me a list of the additional historical information you would obtain.”

Prompt 2:

“You indicated you would ask the patient about X. Why is this important to you?”

**ASK THIS PROMPT TWICE ABOUT TWO SEPARATE TOPICS**
Examples: Which over-the-counter medication? Quantity/dose/frequency/duration of medication? Associated symptoms (fever, diarrhea, jaundice, etc)? Social history (alcohol and substance use)?

Scoring Guidelines:

Rationale: Learner should ask questions to obtain further details about the abdominal pain (location, quality, duration) and any associated symptoms. They should also elicit details regarding the over-the-counter medication, and pertinent history regarding PMH/PSH and social history.

General Guidelines:

- If candidate begins managing the case like a standard case, examiner states, “Remember Doctor, there is no role playing in this case. Please list the additional information you want to obtain.”
- If candidate does not offer a complete list of historical information, examiner should pause long enough to allow them to list additional items, before asking “why” questions.
- If candidate mentions “past medical history,” or “social history,” examiner clarifies by asking, “What specifically do you want to know about PMH/social history?”

**PHYSICAL EXAMINATION:**

“You are provided with the following additional historical information:”

1. Patient drinks alcohol infrequently. No intravenous drug use.
2. No recent travel history
3. She has no history of gallbladder disease.
4. There has been no melena or hematemesis.
5. She has been taking acetaminophen for her migraine for several days. She is unsure the exact amount but when the headache got severe, she took a large handful as she just wanted the pain to go away. She has taken many large handfuls every day for the last five days.

Prompt 3:

“Based on what you now know, please give me a list of specific physical examination findings you would be looking for.”

Prompt 4:

“Doctor, you examined X during the physical exam. Please explain how that would help you.”

Examples: Assess location of tenderness on abdominal exam, signs of dehydration, and peritoneal signs, presence of jaundice

Scoring Guidelines:

Rationale: Learner should examine the patient with a focus on the abdominal exam (location of tenderness, presence of rebound or guarding, and evaluating for signs of liver injury (presence of skin jaundice, scleral icterus) given the acetaminophen ingestion history. They should also evaluate for signs of dehydration given tachycardia and history of vomiting (skin turgor, capillary refill, dry mucous membranes).

**DIFFERENTIAL DIAGNOSIS**

“You are provided with the following physical exam findings:”

- **General Appearance:** Awake, alert, appears uncomfortable on exam.
- **Vital Signs:** T 98.6° F Oral, BP 115/75, HR 114, RR 18, Pulse Ox: 100% on RA.
- **Head:** Normocephalic, atraumatic.
- **Eyes:** Icteric sclera, pupils are equal, round, reactive to light and accommodation (PERRLA) (4 mm to 3 mm).
- **Ears:** Normal external exam, normal auditory canal and timpanic membrane (TM).
- **Mouth/Throat:** Dry mucous membranes.
- **Neck:** Supple, full range of motion, no cervical lymphadenopathy.
- **Skin:** Jaundiced skin, no rashes.
- **Lungs:** Clear to auscultation bilaterally, good air movement.
- **Heart:** Tachycardic rate and rhythm, no murmurs, rubs or gallops.
- **Abdomen:** TTP in RUQ, no rebound or guarding, negative Murphy’s sign, stool negative for occult blood.
- **Extremities:** Good pulses, no clubbing, cyanosis, or edema.
- **Neurological:** Alert, oriented to person, place, time (AOX3), cranial nerves II–XII normal, strength 5/5 all four extremities, normal sensation, normal cerebellar exam, normal reflexes.
- **PSYCH**: Normal affect and mood, no suicidal ideations.

Prompt 5:

“Based on what you now know, what are the top three items on your differential diagnosis based on the most likely conditions?” (If more than three conditions are mentioned, say, “OK thank you. Please give me your three, and only three, most likely diagnoses.”)

Example of appropriate differential diagnoses include:

- Acetaminophen toxicity
- Viral hepatitis
- Hepatobiliary disease
- Alcohol hepatitis
- Pancreatitis
- Autoimmune hepatitis
- Budd-Chiari syndrome
- Pyogenic liver abscess
- Retrocecal appendicitis
- Fitz-Hugh Curtis syndrome

Scoring Guidelines:

Rationale: Mentioning any diagnoses above, pathologies related to liver, gastric, gallbladder, or pancreas.

**DIAGNOSTIC STUDIES**

Prompt 6:

“Based on what you know and your working differential diagnosis, what, if any, diagnostic studies would you order?”

Prompt 7:

“Doctor, you ordered X. Why X?”

**ASK THIS PROMPT TWICE ABOUT TWO SEPARATE TOPICS**

Scoring Guidelines:

Rationale: Diagnostic studies evaluating for the above differential diagnosis: liver, gastric, gallbladder, pancreas pathologies. Also diagnostic studies investigating ingestions, both the known acetaminophen ingestion and possible other co-ingestions.

*After the candidate responds*,

***PROVIDE STIMULUS 2, 3, 4, 5, 6, or 7, depending on what the learner ordered.

**TREATMENT AND OTHER ACTIONS**

Prompt 8:

“Based on what you now know, what treatments, if any, would you order and/or what actions, if any, would you perform?”

Prompt 9:

“Doctor, you ordered X. Why X?”

**ASK THIS PROMPT TWICE ABOUT TWO SEPARATE TOPICS**
Examples: “Doctor, you ordered intravenous fluids (IVFs), why IVFs? Doctor, you ordered N-Acetylcysteine (NAC), why NAC?”

Scoring Guidelines:

Rationale: NAC for elevated acetaminophen level and abnormal liver function tests (LFTs) (chronic ingestions so unable to use Rumack-Matthews nomogram)

Intravenous fluids (IVFs) for tachycardia and dry mucous membranes on exam

**FINAL DIAGNOSIS**

Prompt 10:

“Based on everything you know about this case, what is your final diagnosis?”

1. Acetaminophen toxicity

Scoring Guidelines:

Rationale: Verbalizing acetaminophen toxicity or acetaminophen overdose meets the critical action. If the candidate mentions something vague such as abdominal pain or liver dysfunction, examiner asks “Can you be more specific about the diagnosis?”

**DISPOSITION**

Prompt 11:

“Based on what you know, what should be the disposition of this patient?”

Prompt 12:

“Why would you [admit/discharge] this patient?”

Scoring Guidelines:

Rationale: Verbalizing an intensive care unit (ICU) level admission for acetaminophen toxicity and explaining it is necessary for close monitoring of liver function, complications, and antidote administration (NAC).

**TRANSITION OF CARE**

Prompt 13:

“What specific actions would you take at the time of [admission/discharge]?

Scoring Guidelines:

Rationale: Verbalizing a final action such as explaining the diagnosis to the patient, signing out care to the intensive care team and contacting poison center or the liver service for further recommendations.

*Thank you, Doctor. That concludes this case.*

*Please tear up your notes.*

## ORAL BOARDS ASSESSMENT

*Acetaminophen Toxicity*

Learner: _________________________________________[Table t2-jetem-10-1-si1]

### Stimulus Inventory

[Table t3-jetem-10-1-si1]

#### Stimulus #1

##### Structured Interview: Patient Information

[Table t4-jetem-10-1-si1] [Table t5-jetem-10-1-si1] [Table t6-jetem-10-1-si1]

#### Stimulus #2

##### CBC

[Table t7-jetem-10-1-si1]

#### Stimulus #3

##### CMP

[Table t8-jetem-10-1-si1]

#### Stimulus #4

##### Coagulation panel

[Table t9-jetem-10-1-si1]

#### Stimulus #5

##### Urine Drug Screen, Serum Alcohol

[Table t10-jetem-10-1-si1] [Table t11-jetem-10-1-si1]

#### Stimulus #6

##### APAP, Salicylate level

[Table t12-jetem-10-1-si1]

#### Stimulus #7

##### Right Upper Quadrant US

No evidence of gallstones. No gallbladder wall thickening. No pericholecystic fluid.
